# RBM20-Mediated Pre-mRNA Splicing Has Muscle-Specificity and Differential Hormonal Responses between Muscles and in Muscle Cell Cultures

**DOI:** 10.3390/ijms22062928

**Published:** 2021-03-13

**Authors:** Rexiati Maimaiti, Chaoqun Zhu, Yanghai Zhang, Qiyue Ding, Wei Guo

**Affiliations:** 1 Animal Science Department, University of Wyoming, Laramie, WY 82071, USA; rmaimait@uwyo.edu (R.M.); cqnzhu@ucdavis.edu (C.Z.); 2 Animal and Dairy Sciences, University of Wisconsin-Madison, Madison, WI 53706, USA; zhang2485@wisc.edu (Y.Z.); qding35@wisc.edu (Q.D.)

**Keywords:** RBM20, pre-mRNA splicing, titin, hormones, skeletal muscle

## Abstract

Pre-mRNA splicing plays an important role in muscle function and diseases. The RNA binding motif 20 (RBM20) is a splicing factor that is predominantly expressed in muscle tissues and primarily regulates pre-mRNA splicing of Ttn, encoding a giant muscle protein titin that is responsible for muscle function and diseases. RBM20-mediated Ttn splicing has been mostly studied in heart muscle, but not in skeletal muscle. In this study, we investigated splicing specificity in different muscle types in Rbm20 knockout rats and hormonal effects on RBM20-mediated splicing both in cellulo and in vivo studies. The results revealed that RBM20 is differentially expressed across muscles and RBM20-mediated splicing is muscle-type specific. In the presence of RBM20, Ttn splicing responds to hormones in a muscle-type dependent manner, while in the absence of RBM20, Ttn splicing is not affected by hormones. In differentiated and undifferentiated C2C12 cells, RBM20-mediated splicing in response to hormonal effects is mainly through genomic signaling pathway. The knowledge gained from this study may help further understand muscle-specific gene splicing in response to hormone stimuli in different muscle types.

## 1. Introduction

Skeletal muscle comprises around 40% of the body weight in vertebrate animals, including humans, and supports multiple bodily functions such as body movement through muscle contraction [1]. The contractile function of the skeletal muscle relies on the contractile unit sarcomere consisting of thick filament (myosin) and thin filament (actin) as well as third filament (titin). Titin is also the third most abundant constituent in skeletal muscle and plays multiple roles in skeletal muscle function and diseases [2]. Currently, titin is the largest protein found in the vertebrate muscles that produces passive tension during muscle contraction [3]. Titin has numerous isoforms resulting from alternative splicing among 363 exons [4,5,6,7]. These isoforms are mainly generated by the extensive alternative splicing in the middle of the immunoglobulin (Ig) region and the PEVK (proline [P], glutamate [E], valine [V], and lysine [K]) domain [8,9,10,11,12]. The Ig region and the PEVK domain are the major components of the titin spring element that generates passive forces when skeletal muscle contracts [13,14,15]. Additionally, titin functions as a biomechanical sensor through interaction with many other proteins binding to titin’s Z-disk, central I-band and M-line regions that can mediate hypertrophic signaling and protein quality control [2,16,17,18]. For instance, titin has a binding site for the RING finger protein called muscle-specific finger protein-1 (MURF1), which is an E3 ubiquitin ligase responsible for controlling protein turnover. A recent study has shown that titin functions as a mechanosensor that regulates muscle hypertrophy [19]. Therefore, alternative titin exon usages and titin size switching resulting from alternative splicing may interrupt mechanosensing signals for muscle function and hypertrophy.

RNA binding protein 20 (RBM20) is a major regulator of alternative splicing of Ttn [20,21,22,23]. It is predominantly expressed in striated muscles with second highest expression level in skeletal muscle [12,18,24]. Deletion mutation of Rbm20 with loss-of-function switches titin isoforms to one single largest isoform in both heart and skeletal muscles [11,20,21,23,25,26,27]. Alternative exons used in Ttn are mainly located in the Z-band region, the middle Ig region and the PEVK region in the I-band and the M-band [11,12,18,21]. A recent study reported that RBM20 regulates pre-mRNA splicing in the Ttn Z-band region only in skeletal muscle, and not in cardiac muscle [24]. Although RBM20 is a major regulator of Ttn splicing, it also regulates pre-mRNA splicing of other muscle genes such as Ca2+/calmodulin-dependent protein kinase (Camk2), Ldb3 and Pdlim3 [20,22,28]. Mis-splicing of these genes in skeletal muscle has been implicated in various kinds of muscle diseases including myotonic dystrophy type 1 and autosomal dominant myofibrillar myopathy [29,30,31,32,33]. These studies show that understanding the mechanisms of RMB20 in skeletal muscle is important for muscle function. Studies in the heart demonstrate that hormones insulin and T3 (triiodothyronine) regulate Ttn splicing through the PI3K/Akt/mTOR signaling pathway (non-genomic signaling pathway) in a RBM20-dependent manner [34,35]. However, it is unclear whether this non-genomic signaling pathway also plays an important role in Ttn splicing in skeletal muscle. In this study, we investigated RBM20 expression levels and RBM20-mediated pre-mRNA splicing regulated by hormones (thyroid and insulin) in fast and slow muscle types, and also determined whether thyroid hormone modulates the RBM20-mediated pre-mRNA splicing via the genomic or non-genomic signaling pathways in differentiated and undifferentiated muscle myoblasts (C2C12 cells).

## 2. Results 

### 2.1. RBM20 Expression Responds to Hormone Differently in Distinct Muscle Types

**Figure 1:**
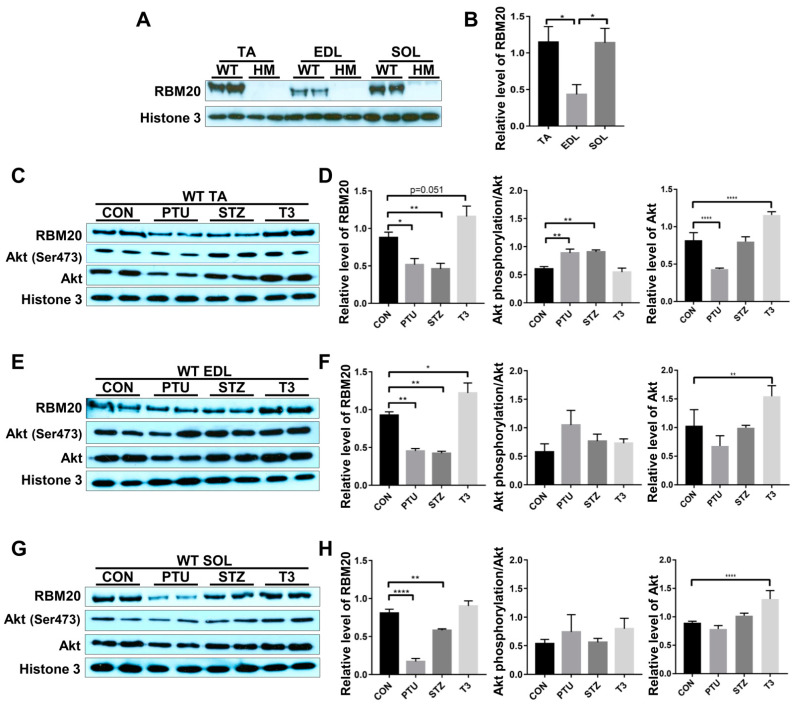
Hormonal effects on RBM20 expression and Akt activity in different muscle types. (**A**,**B**) RBM20 expression in the TA, EDL, SOL muscles of WT and HM rats. (**C**,**D**) RBM20 expression and Akt activity in the WT TA muscle treated with PTU, STZ and T3; (**E**,**F**) RBM20 expression and Akt activity in the WT EDL muscle treated with PTU, STZ and T3; (**G**,**H**) RBM20 expression and Akt activity in the WT SOL muscle treated with PTU, STZ and T3. WT, wildtype; HM, RBM20 homozygous knockout; CON, control; PTU, propylthiouracil; STZ, streptozocin; T3, trriodothyronine; TA, tibialis anterior; EDL, extensor digitorum longus; SOL, soleus; Histone 3, protein loading control. Mean ± SEM (*n* = 4), * *p* < 0.05, ** *p* < 0.01, **** *p* < 0.0001.

RBM20 is a master regulator of Ttn splicing in muscle tissues [20,21]. RBM20-mediated Ttn splicing through hormone stimuli has been studied in heart muscle [34,35], but little is known about it in skeletal muscles. In particular, RBM20 expression and RBM20-mediated Ttn splicing have not been well assessed in different muscle types. In this study, we examined Ttn splicing pattern and RBM20 expression levels in different muscles and determined how hormones (thyroid and insulin) modulate Ttn splicing through RBM20 expression in skeletal muscles. We first evaluated RBM20 expression level using Western blotting in tibialis anterior (TA, a fast and slow mixed muscle), extensor digitorum longus (EDL, fast type) and soleus (SOL, slow type) muscles. In Rbm20 homozygous knockout (HM) muscles, there is no RBM20 expression in all three type of muscles, while in wildtype (WT) muscles, TA and SOL muscles express significantly higher RBM20 level than that in EDL muscle (Figure 1A,B).

Thyroid and insulin have been shown to regulate Ttn splicing in heart muscle via the PI3K/Akt signaling pathway, a non-genomic pathway [34,35]. Next, we tested whether these hormones also regulated RBM20 expression via the PI3K/Akt signaling pathway in skeletal muscles. We observed that RBM20 expression was significantly decreased in all three muscles treated with PTU and STZ, and increased with T3 treatment (Figure 1C–H). In the TA muscle, Akt was activated with PTU and STZ treatment, but not with T3 treatment. Lower T3 level decreased Akt expression level, while higher T3 level increased Akt expression level (Figure 1C,D). In the EDL and SOL muscles, Akt signaling was not activated with all treatments, but Akt expression level was increased by T3 treatment (Figure 1E–H). These data suggest that RBM20 is differentially expressed in different muscle types, with higher expression levels in the SOL and TA muscles than that in the EDL muscle. Decreased hormone levels by PTU and STZ suppress RBM20 expression in all muscles, while increased thyroid level elevates RBM20 expression significantly in the EDL muscle, but not in the TA and SOL muscles. However, RBM20 level tends to be higher in TA muscle with T3 treatment. Unexpectedly, the Akt signaling seemed to be blunted to all treatments in the fast (EDL) and slow (SOL) muscles even though Akt expression was increased with T3 treatment. The TA muscle shows the activated Akt with lower level of thyroid and insulin, but no change with increased thyroid level as well as decreased Akt level in PTU treatment and increased Akt level in T3 treatment.

### 2.2. Titin Isoform Switching Has Differential Response to RBM20 Level and Hormones in Different Muscle Types

**Figure 2:**
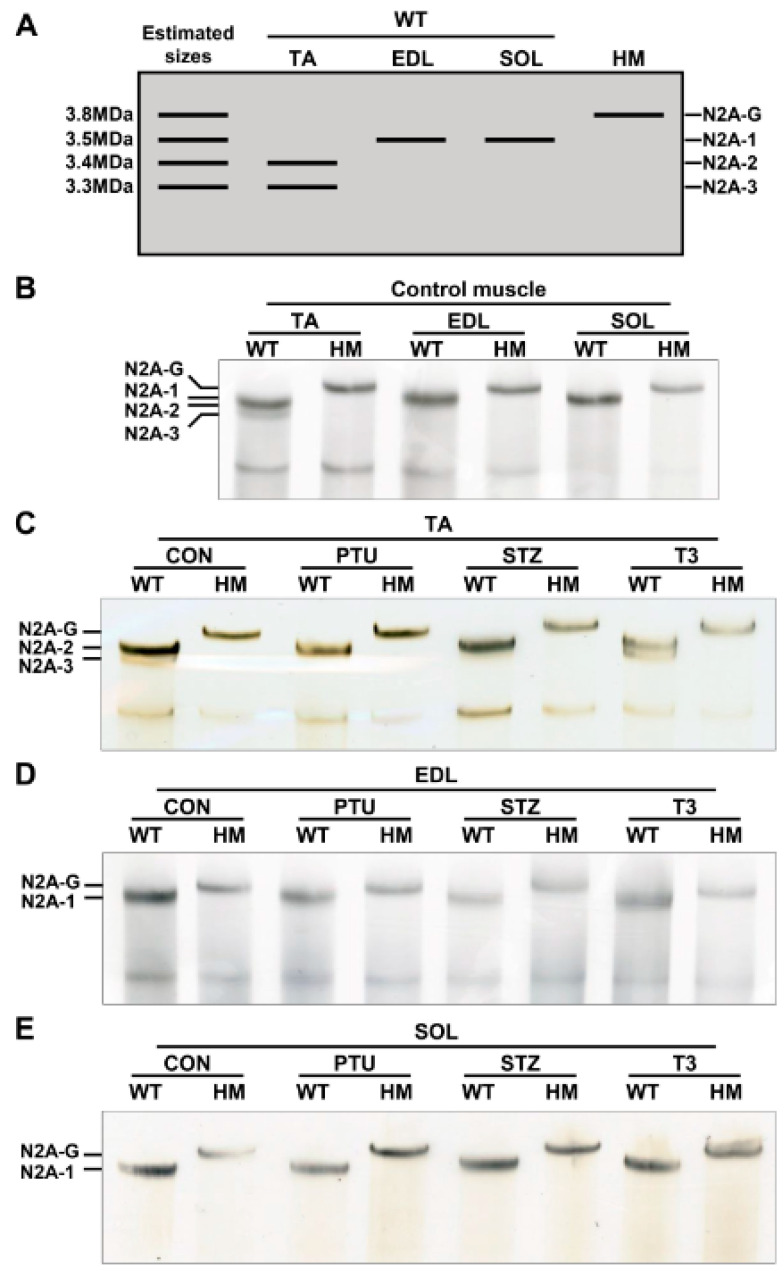
Hormonal effects on titin isoform switching in different muscle types. (**A**) Schematic diagram of titin isoforms and estimated sizes in skeletal muscle; (**B**) Titin isoform switching in different muscle types between WT and HM without treatment; (**C**–**E**) Titin isoform switching in different muscle types between WT and HM with PTU, STZ and T3 treatment respectively. WT, wildtype; HM, RBM20 homozygous knockout; CON, control; PTU, propylthiouracil; STZ, streptozocin; T3, triiodothyronine; TA, tibialis anterior; EDL, extensor digitorum longus; SOL, soleus; N2A-G, giant isoform; N2A 1-3, titin isoforms; *n* = 3 for all treated and untreated muscles.

Our previous studies indicated that adult heart muscle expresses larger titin isoforms when RBM20 is down-regulated, and vice versa [20,21,27]. Thyroid or insulin up-regulates RBM20 expression, and thus, switches titin to smaller isoforms in adult heart muscle [34,35]. It is unclear whether skeletal muscle follows this transition pattern or not. We first detected titin isoforms in TA, EDL and SOL muscles using 1% agarose gel electrophoresis. With higher RBM20 expression in TA muscle (Figure 1A), titin appears as two bands with sizes of approximately 3.3 MDa for the lower band (N2A-3) and 3.4 MDa for the intense upper band (N2A-2) (Figure 2A,B). In contrast, with lower RBM20 levels in EDL muscle (Figure 1A), titin shows one band (N2A-1) with a size of approximately 3.5 MDa, which is slightly larger than the N2A-2 band in the TA muscle (Figure 2A,B). We anticipate that titin could be expressed as a smaller isoform in the SOL muscle with higher RBM20 expression, as observed in heart muscle. Unexpectedly, it shows a larger titin isoform similar to that in the EDL muscle with lower RBM20 levels (Figure 2A,B). Furthermore, in the absence of RBM20, all three muscles express the same single largest titin isoform (N2A-G), with a size of approximately 3.8 MDa when compared to WT (Figure 2A,B). These results imply that the TA and EDL muscles follow the switching pattern as indicated in heart muscle, but not in the SOL muscle.

Next, both WT and HM rats were treated with PTU and STZ to reduce thyroid and insulin levels, respectively, and with T3 to increase the thyroid hormone level. Titin was then detected in TA, EDL and SOL muscles. In the TA muscle, PTU and STZ treatments completely switched the smaller N2A-3 to the larger N2A-2 isoform, while T3 treatment decreased the level of larger isoform N2A-2 and increased the level of the smaller N2A-3 isoform when compared to the control group (Figure 2C). There no change was observed in HM muscles between control and treated groups (Figure 2C). Furthermore, there were no changes observed in the EDL and SOL muscles of WT rats, or in HM rats with any of the treatments. In WT EDL and SOL muscles, the same-sized titin isoform N2A-1 was expressed across all treatments, but smaller than N2A-G in HM EDL and SOL muscles (Figure 2D,E). These results reveal that the hormonal effect on titin isoform switching in TA muscle is similar to that in heart muscle. Conversely, the hormonal effect on titin isoform switching in EDL and SOL muscles is limited, suggesting RBM20-mediated Ttn splicing through hormones is differentially regulated in different muscle types.

### 2.3. Ttn Exons Are Alternatively Used with Hormone Treatment in Different Muscle Types

**Figure 3:**
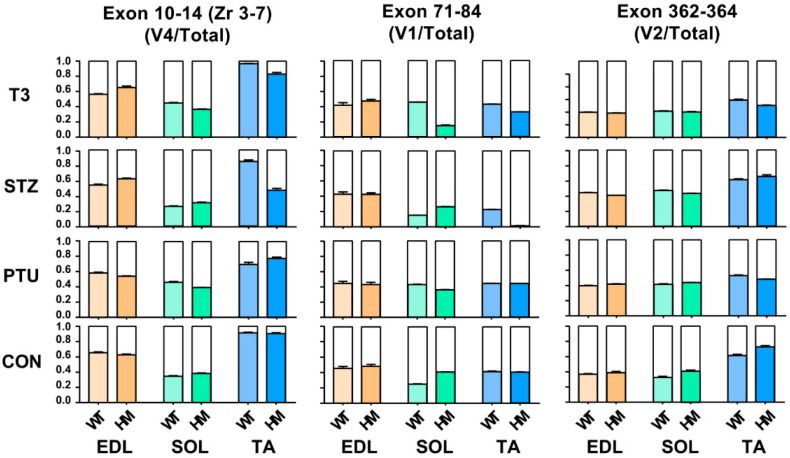
Hormonal effects on titin major splice variants in Z-, I- and M-bands between WT and HM across different muscles. WT, wildtype; HM, RBM20 homozygous knockout; CON, control; PTU, propylthiouracil; STZ, streptozocin; T3, trriodothyronine; TA, tibialis anterior; EDL, extensor digitorum longus; SOL, soleus.

Titin is the largest protein identified in vertebrate animals, with a molecular weight from approximately 3 to 4 MDa resulting from alternative pre-mRNA splicing [36]. In terms of titin size, it is almost impossible to distinguish the subtle changes based on current gel electrophoresis technology. Hence, the above observation, whereby titin size exhibits no change in EDL and SOL muscles of WT rats between treated and untreated groups, does not mean that Ttn exons are not alternatively used, because small number of exon usages may not change overall titin size. To determine whether Ttn exons are still alternatively used without alteration of overall titin size through hormone treatment, RT-PCR was performed in known Ttn splicing regions: Z-band, I-band and M-band. Primers used in this study were designed against known alternative exons [11,24] (Figure S1A and Table S1). Band densitometry was quantified by using the ratio of major band to total band density. Only validated variants were used in this study based on our previous report [24] which are labelled in the gel images. In the titin Z-band region, the splicing pattern remained the same in both treated and untreated EDL, SOL and TA muscles of WT and HM rats, respectively, with primers spanning exons 7 to 10 (Zr1-3) (Figure S2A–C). However, with primers spanning exons 10 to 14 (Zr3-7), we observed that the ratio of the variant 4 (v4) to total major variants was decreased in WT EDL muscle with all treatments, and in HM EDL muscle only with PTU treatment; the ratio of v4 to total major variants increased in WT SOL muscle with PTU and T3 treatment, but decreased in HM SOL muscle with STZ treatment; the ratio of v4 to total major variants was significantly increased in WT TA muscle with T3 treatment, and decreased in HM TA muscle with all treatments (Figure 3 and Figure S1B–E).

We then assessed exon usages in the I-band with primers designed against middle Ig region (Exon 71-84) [11,24] (Figure S1A and Table S1). In the middle Ig region (exon 71-84), we revealed that the ratio of the major variant 1 (v1) to total variants (v1+v2) stayed the same in both WT and HM EDL muscles with all treatments (Figure 3 and Figure S1F,I). The ratio of v1 to total variants increased in WT SOL muscle with PTU and T3 treatment, but decreased with STZ treatment, while the ratio was decreased in HM SOL muscle with all treatments compared to the control HM (Figure 3 and Figure S1G,I). In TA muscle, the ratio increased in WT treated with PTU and T3 and decreased in WT with STZ treatment, while the ratio increased in HM with PTU treatment, and decreased in HM with STZ and T3 treatment (Figure 3 and Figure S1H,I). Lastly, we examined exon usages in the M-band region with primers against exon 362-364 [11,24]. The results demonstrated that the ratio of the major variant 2 (v2) to total variants exhibited no change in WT EDL muscle with PTU treatment, but increased with STZ and T3 treatment; the ratio exhibited no change in HM EDL muscle with STZ and T3 treatment, but increased with PTU treatment (Figure 3 and Figure S1J,M). In the SOL muscle, the ratio of v2 to total variant increased in WT with all treatments, but no change was observed in HM with any of the treatments when compared to control (Figure 3 and Figure S1K,M). In the TA muscle, the ratio was decreased in WT with PTU treatment, and exhibited no changes in WT with STZ and T3 treatment, while the ratio was significantly decreased in HM with all treatments (Figure 3 and Figure S1L,M). Taken together, the RT-PCR results show that although the titin size change is undetectable with current gel electrophoresis technology, Ttn exons are still alternatively used through treatment of hormones.

### 2.4. Splicing Pattern of CamkIId and CamkIIg between WT and HM in Response to Hormones in Different Muscle Types

**Figure 4:**
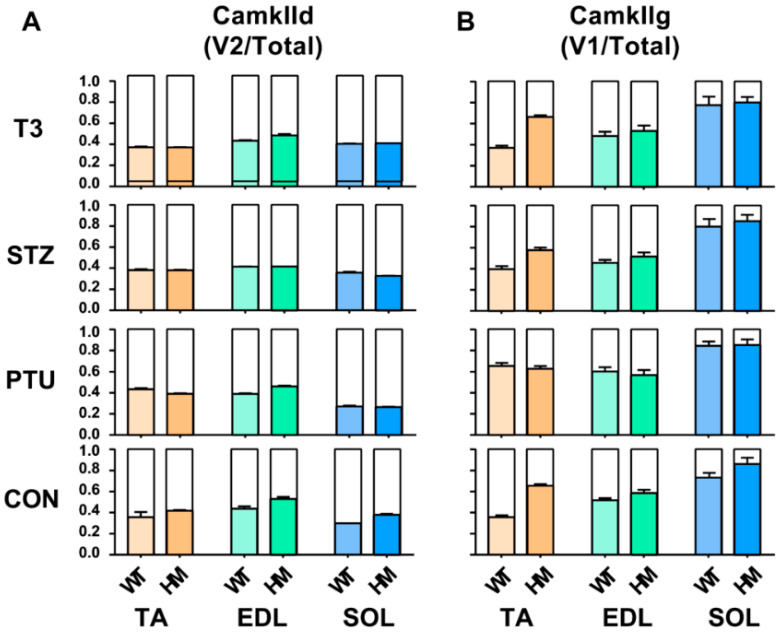
Hormonal effects on CamkIId and CamkIIg major splice variants between WT and HM across different muscles (**A**,**B**). WT, wildtype; HM, RBM20 homozygous knockout; CON, control; PTU, propylthiouracil; STZ, streptozocin; T3, trriodothyronine; TA, tibialis anterior; EDL, extensor digitorum longus; SOL, soleus.

In addition to Ttn splicing, RBM20 also regulates the splicing of other genes such as CamkIId and CamkIIg, which have been validated in cardiac muscle [20,22,23]. These two genes have been found in skeletal muscle. Here, we further determined the hormonal effects on splicing pattern of these two genes in skeletal muscle. We first determined the hormonal effect on splicing pattern of CamkIId. In the TA muscle, the ratio of the major variant 2 (v2) to total variants increased in WT treated with PTU, and had no changes in WT treated with STZ and T3. The ratio maintained the same in HM with all treated groups by compared to control group (Figure 4A and Figure S3A,B). In the EDL muscle, the ratio was not changed in WT with all treatments, but decreased in HM with PTU and STZ treatment and had no change in HM with T3 treatment (Figure 4A and Figure S3C,D). In the SOL muscle, the ratio decreased in WT treated with PTU and increased with STZ and T3, while the ratio decreased in HM treated with PTU and STZ and increased with T3 (Figure 4A and Figure S3E,F). Next, we determined the hormonal effect on splicing pattern of CamkIIg in skeletal muscle. In the TA muscle, the larger variant v1 increased in control HM group when compared to WT group, and this was also found in the WT and HM group treated with STZ and T3, respectively. The larger variant v1 increased in the WT group treated with PTU, but had no change in WT treated with STZ and T3. The larger variant v1 had no change in HM groups with all treatments by compared to control HM group (Figure 4B and Figure S3G,H). In the EDL muscle, we also observed an increased level of the larger v1 in HM control and STZ treatment groups compared to WT control and STZ treatment group, respectively. The larger v1 increased in WT treated with PTU and decreased in WT treated with STZ compared to WT control. However, the larger v1 had no changes in all HM treated groups compared to the HM control group (Figure 4B and Figure S3I,J). In the SOL muscle, none of the treatments impacted the splicing pattern of CamKIIg (Figure 4B and Figure S3K,L). These results indicate that hormonal effects on pre-mRNA splicing of CamkIId and CamkIIg genes are muscle-type specific.

### 2.5. RBM20-Mediated Pre-mRNA Splicing Is Regulated via Both Genomic and Non-Genomic Signaling Pathways, with More Profound Effect by Genomic Pathways in Undifferentiated C2C12 Myoblasts

**Figure 5:**
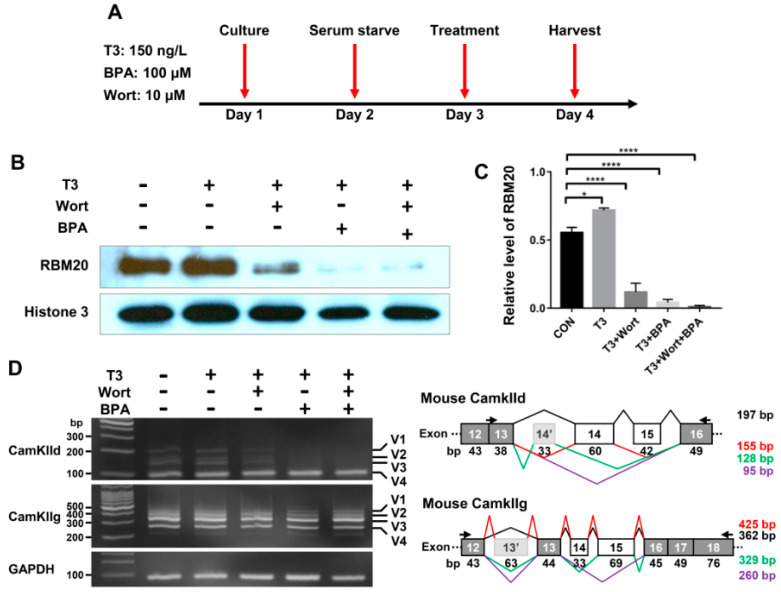
RBM20 expression and pre-mRNA splicing of CamkIId and CamkIIg in undifferentiated C2C12 cells via the genomic and non-genomic pathways. (**A**) Treatment timeline of C2C12 cells with T3, Wort and BPA; (**B**) Western blotting of RBM20 expression in C2C12 cells treated with T3, Wort and BPA respectively; (**C**) Quantification of RBM20 expression level. (**D**) RT-PCR detection of pre-mRNA splicing of CamKIId and CamKIIg genes in C2C12 cells treated with T3, Wort and BPA respectively. CON, control; T3, trriodothyronine; Wort, Wortmannin; BPA, Bisphenol A; V1-4, splicing variants of CamkIId and CamkIIg; Histone, protein loading control. Mean±SEM (*n* = 5), * *p* < 0.05, **** *p* < 0.001.

It has been reported that thyroid hormone has both genomic and non-genomic actions [37,38,39,40]. To investigate the thyroid-activated signaling in the regulation of pre-mRNA splicing via RBM20 in skeletal muscle, we cultured C2C12 cells to evaluate the impact of non-genomic and genomic pathways activated by thyroid hormone on RBM20-mediated pre-mRNA splicing. The inhibitor Wortmannin inhibits non-genomic signaling through the PI3k/Akt signaling pathway, while the inhibitor BPA represses the genomic pathway by acting as an antagonist against T3 [41,42,43,44,45]. To avoid the interference with serum factors, undifferentiated C2C12 cells were serum-starved for 24 h and then were treated with T3, T3+Wortmannin, T3+BPA and T3+Wortmannin+BPA respectively for another 24 h (Figure 5A). Harvested cells were lysed for protein and RNA preparation. Proteins were used for Western blotting analysis using antibody against RBM20. T3 treatment significantly increased RBM20 level when compared to untreated control group, while treatment with T3+Wortmannin, T3+BPA and T3+Wortmannin+BPA remarkably decreased RBM20 expression. Interestingly, with BPA addition and both BPA and Wortmannin addition, RBM20 expression was almost eliminated (Figure 5B,C). Next, we examined the impact of non-genomic and genomic pathways on splicing pattern of two RBM20 target genes, CamkIId and CamkIIg. We discovered that the non-genomic pathway inhibited by Wortmannin did not change the splicing pattern of CamkIId, but decreased the level of the larger variants (v1-3) of CamkIId slightly. However, inhibition of the genomic pathway with BPA supplement dramatically altered the splicing pattern of CamkIId, and diminished the larger variants (v1-3). Unexpectedly, neither non-genomic nor genomic pathways had an effect on the splicing pattern of CamkIIg (Figure 5D). To test whether longer treatment impacted splicing pattern through both non-genomic and genomic pathways, we extended our treatment to 48 h in undifferentiated C2C12 cells and observed the same effect as for 24 h treatment with BPA on the splicing pattern of CamkIId and CamkIIg (Figure S4A–D). These results suggest that RBM20 expression and RBM20-mediated pre-mRNA splicing are regulated by both genomic and non-genomic pathways with more profound effect by genomic pathway in undifferentiated C2C12 myoblasts.

### 2.6. Similar Effect of Genomic and Non-Genomic Pathways on RBM20 Expression and RBM20-Mediated Pre-mRNA Splicing in Differentiated C2C12 Cells

**Figure 6:**
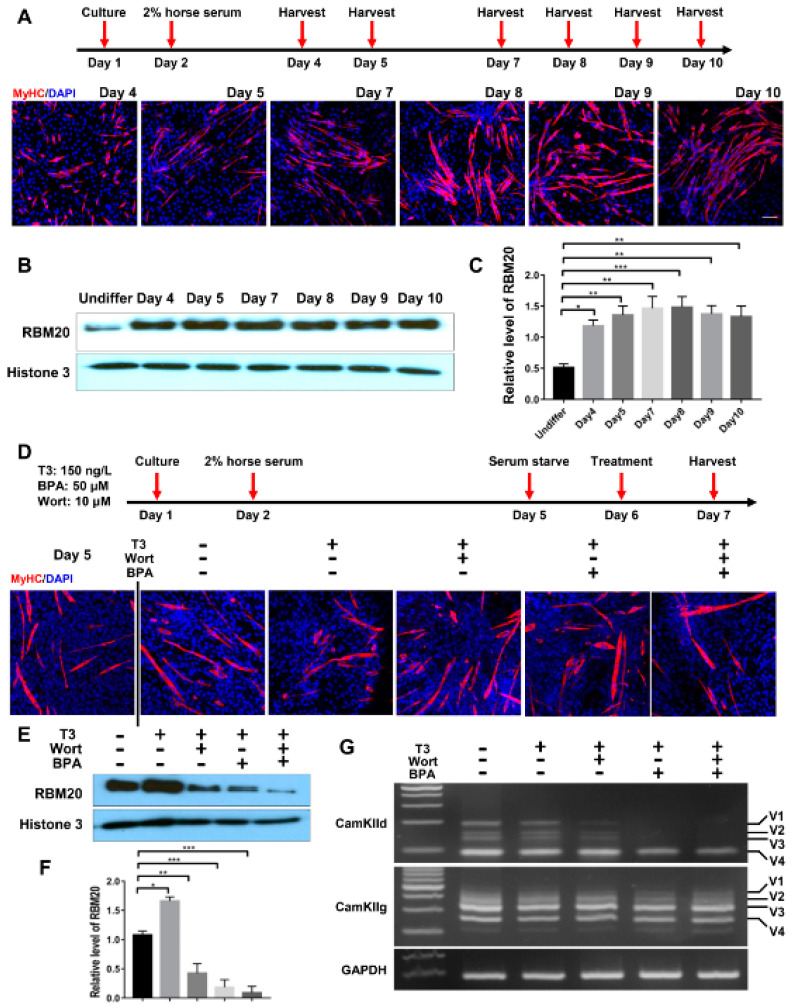
RBM20 expression and gene splicing of CamkIId and CamkIIg in differentiated C2C12 cells via the genomic and non-genomic pathways. (**A**) Cell harvest timeline and Immunofluorescent staining of myosin heavy chain during C2C12 differentiation; (**B**,**C**) RBM20 expression and quantification during C2C12 differentiation. (**D**) Differentiation and treatment timeline of C2C12 cells with T3, Wort and BPA respectively and immunostaining with myosin heavy chain antibody during C2C12 differentiation; (**E**,**F**) Western blotting of RBM20 expression and quantification in differentiated C2C12 cells. (**G**) RT-PCR detection of pre-mRNA splicing of CamKIId and CamKIIg genes in differentiated C2C12 cells. CON, control; T3, trriodothyronine; Wort, Wortmannin; BPA, Bisphenol A; Undiffer, undifferentiation; Histone, protein loading control; GAPDH, housekeeping gene; V1-4, splicing variants of CamkIId and CamkIIg. Mean ± SEM (*n* = 5), * *p* < 0.05, ** *p* < 0.01; *** *p* < 0.001.

To assess whether the in cellulo differentiated myofibers alter RBM20 expression and RBM20-mediated pre-mRNA splicing via non-genomic and genomic pathways, we differentiated the C2C12 to mature myofibers and determined the pre-mRNA splicing of two RBM20 target genes CamkIId and CamkIIg again. C2C12 cells were differentiated for 8 days and differentiated myofibers were identified through immunostaining against MyHC antibody (Figure 6A). Western blotting results showed that RBM20 was significantly increased in differentiated myofibers at day 4 by compared to undifferentiated C2C12 cells and maintain the high expression level to day 10 (Figure 6B,C). To further investigate whether non-genomic and genomic pathways regulate pre-mRNA splicing through RBM20 in myofibers, we used the differentiated myofibers at day 5 to be treated with T3, T3+Wortmannin, T3+BPA and T3+Wortmannin+BPA for 24 h, respectively, after serum starvation (Figure 6D). We found that T3 addition significantly promoted RBM20 expression, while Wortmannin supplement repressed RBM20 expression with further repression by BPA addition (Figure 6E,F). Next, we determined the splicing pattern of CamkIId and CamkIIg using RT-PCR. The results were similar to the undifferentiated cells. Wortmannin supplement did not change the splicing pattern of CamkIId, but slightly decreased the level of larger variants (v1-3), whereas BPA addition abolished the larger variants (v1-3) (Figure 6G). However, Wortmannin and BPA addition did not alter splicing pattern of CamkIIg (Figure 6G). These results revealed the similar impact of genomic and non-genomic signaling pathway on RBM20 expression and RBM20-mediated pre-mRNA splicing to the undifferentiated C2C12 cells. Furthermore, whether this in cellulo observation occurs in vivo needs be explored in future studies.

## 3. Discussion

Contractile proteins and cytoskeletons in skeletal muscle enable muscle contraction [46]. Genes encoding these proteins may undergo alternative splicing during prenatal and postnatal stages as well as under environmental changes [47,48,49]. Alterations of these splicing events caused by genetic mutations and environmental changes and so forth have been associated with various skeletal muscle growth and pathology [50,51,52]. Our current study revealed that changes of thyroid and insulin hormone levels regulate muscle gene alternative splicing through RBM20 in different muscle types.

Our data indicate that splicing factor RBM20 is differentially expressed in different skeletal muscle types. Fast muscle (EDL) expresses a lower level of RBM20 compared to slow (SOL) and slow/fast mixed (TA) muscles. According to previous studies in heart muscle, higher levels of RBM20 in adult hearts favor smaller titin isoform expression, whereas lower levels of RBM20 in adult hearts favor larger titin isoform expression [20,21]. As a result, we expect that decreased expression of RBM20 in fast muscle presents a larger titin isoform than those in slow and mixed muscles with higher RBM20 levels. We did observe a larger titin isoform expressed in fast muscle (EDL); however, unexpectedly, slow muscle (SOL) also expressed a larger titin isoform of similar size to the fast muscle. The fast and slow mixed muscle (TA) appeared as two isoforms, one with larger size and one with smaller size. Furthermore, the depletion of RBM20 in all of these muscles led to a same single largest titin isoform, no matter what the muscle type was. These results suggest that RBM20 is an indispensable splicing factor for Ttn splicing with unknown regulatory mechanism in distinct muscle types. Titin’s elasticity properties play a key role in contractile function of muscle tissues. The variability of titin size contributes to passive stiffness of different muscle types [2,15,18]. Titin isoform switching beyond its normal size may cause abnormal muscle contractile function and disease [2]. In addition, Titin also acts as a protein binding hub for hypertrophic signaling and mechanosensing signaling [7,17]. Titin alternative exon usages may also affect titin–protein interaction, and thus the signaling transduction for downstream gene expression. The knowledge gained from this study may help understand the relationship between titin size change and muscle function in future studies.

On the other hand, due to the giant size of titin and the limitation of current gel electrophoresis technology, the subtle changes of the giant protein size are not able to be detected. To determine whether Ttn exons were still being alternatively used, we performed RT-PCR in mostly splicing-favorable regions of Ttn. We observed that even though hormone treatment did not change the overall size of titin, Ttn exons were still alternatively used in different muscle types. In addition to Ttn pre-mRNA splicing, we also determined the alteration of splicing pattern in other genes such as CamkIId and CamkIIg which are also the splicing target of RBM20 validated in heart muscle [20,23]. The splicing pattern of these two genes was changed by hormone treatment in heart muscle [34,35]. In skeletal muscles, the results revealed that the splicing pattern of these two genes is also influenced by hormones in a muscle type-specific manner. Early reports indicated that mis-splicing of these two genes induces myopathies in skeletal muscle [29,30,31,32,33]. Therefore, whether loss-of-function of RBM20 in skeletal muscle leads to myopathies needs be investigated in future studies.

Lastly, our previous studies reported that in heart tissue, thyroid and insulin regulate RBM20-mediated pre-mRNA splicing via a non-genomic signaling pathway [34,35,53], i.e., the PI3K/Akt signaling pathway. The current study in skeletal muscle demonstrated that non-genomic signaling pathway seems to be activated differently in distinct muscle fiber types. Additionally, skeletal muscle responds to thyroid hormones to activate the PI3K/Akt signaling very differently in distinct muscle types. One possible explanation for this could be that skeletal muscle is a more plastic tissue that may respond to thyroid hormone treatment differently due to the expression or splicing forms of iodothyronine deiodinases (DIOs) in skeletal muscles [54,55]. Whether RBM20 also regulates the pre-mRNA splicing of DIOs remains unknown. Another possible explanation could be the duration of the treatment time in rats which may induce compensatory or blunted effects of non-genomic signaling pathway in response to long-term exposure of PTU, STZ and/or T3. In this study, all animals were treated over two weeks, which may imply potential blunted effects. Interestingly, studies with differentiated and undifferentiated C2C12 cells in cellulo demonstrate that RBM20 expression is regulated by both non-genomic and genomic signaling pathways, with a profound effect of genomic signaling pathways. Pre-mRNA splicing of CamkIId is significantly regulated via genomic signaling pathways, but not CamkIIg. Even though RBM20 is one of the splicing regulators for CamkIId, this study still cannot rule out the influence of other splicing factors on hormone-induced splicing in skeletal muscle.

In summary, our study revealed that RBM20 expression in skeletal muscle is muscle type dependent. Pre-mRNA splicing of the Ttn gene, a major substrate of RBM20, seems also to be muscle type dependent, meaning RBM20 expression level is not a primary control in some muscle types, for instance, in soleus muscle. These results suggest that other primary mechanism(s) may exist in Ttn pre-mRNA splicing in certain muscle types. Potential mechanism(s) could be posttranslational modification of RBM20 [56,57], protein shuttling and aggregation of RBM20 [56,57], and cooperation with other splicing factors. Hormones also play an important role in RBM20 expression and RBM20-mediated pre-mRNA splicing. Our results imply that both genomic and non-genomic signaling pathways regulate RBM20 expression, and thus RBM20-mediated pre-mRNA splicing in cultured muscle cells with enhanced effect via genomic pathway. This study showed that RBM20-mediated pre-mRNA splicing in skeletal muscle is more complicated than in heart muscle, and further studies are needed to unravel the splicing mechanisms regulated by RBM20 in different skeletal muscle types.

## 4. Materials and Methods

### 4.1. Experimental Animals and Tissues

This study was conducted by using wildtype (*Rbm20*^+/+^, WT) and homozygous knockout (*Rbm20*^-/-^, HM) rats. All the rats were crosses of Sprague-Dawley (SD) X Brown Norway (BN) [20,26]. All strains were originally from Harlan Sprague Dawley, Indianapolis, IN. Animal maintenance and all the experimental procedures was approved by the Institutional Animal Use and Care Committee of the University of Wyoming. Hypothyroidism and hyperthyroidism animal models through treatment with propythiouracil (PTU) and triiodothyronine (T3) in both WT and HM rats were described in our previous publication [34]. A diabetic animal model was made through injection of streptozotocin (STZ) in both WT and HM rats. The detailed procedure was described in our previous publication [35]. All animals used in this study were three months old. Skeletal muscle tissues, soleus (SOL) and tibialis anterior (TA) and extensor digitorum longus (EDL) were dissected and snap-frozen in liquid nitrogen and stored at −80 °C for later use. Protein samples were prepared by homogenizing skeletal muscle tissues in urea-thiourea buffer (8 M urea, 2 M thiourea 75 mM DTT, 3% SDS, 0.05% bromophenol blue, 0.05 M Tris, pH 6.8) as previously described [58].

### 4.2. Western Blotting

Total proteins prepared from above were resolved on SDS-PAGE gel, and electrotransferred to PVDF membrane. Approximate molecular weight of the proteins were determined by comparison with the migration of protein standards (#1610374, Bio-Rad, Hercules, CA, USA). PVDF membrane was probed with primary antibody overnight at 4 °C and incubated with horseradish peroxidase (HRP) secondary antibody for 1 hour at room temperature after washing. The membranes were developed with enhanced chemiluminescence substrate (#1705060, Bio-Rad, Hercules, CA, USA) and the signals were detected by exposing to X-ray films. Quantification was performed based on band densitometry with NIH ImageJ by normalizing to the loading control. Primary antibodies used in this study were histone 3 (#4499S, Cell Signaling Technology, Danvers, MA, USA), Phospho-Akt (Ser473) (#9271, Cell Signaling Technology, Danvers, MA, USA), Akt (#9272, Cell Signaling Technology, Danvers, MA, USA) and home-made RBM20 antibody as described in our previous publications [20].

### 4.3. Titin Gel Electrophoresis

Detection of titin isoforms using titin gel electrophoresis was performed by following the procedure as previously described [58,59].

### 4.4. RT-PCR and DNA Gel Electrophoresis

Total RNAs were isolated from skeletal muscles (TA, EDL and SOL) with Trizol reagent (#T9424, MilliporeSigma, Burlington, MA, USA) and further treated with DNase I (#M6101, Promega, Madison, WI, USA) to remove genomic DNA. Reverse transcriptase assay was conducted with ImProm-II reverse transcription system (#A3801, Promega, Madison, WI, USA). PCR was carried out using a C1000 thermal cycler. Primers for the amplification of the Z- and the M-bands, Exon 71-84 and Exon 156-226 splicing were obtained from previous publications [24,25]. Final PCR production was analyzed using DNA gel electrophoresis and the gel images were obtained by ChemiDoc Imaging System. The band intensity of the gels was quantified by NIH ImageJ. All the primer pairs are provided in Supplementary Table S1.

### 4.5. C2C12 Cultures and Differentiation

C2C12 cells were used for the in cellulo experiments. Undifferentiated C2C12 cells were maintained in DMEM-high-glucose medium supplemented with 20% fetal bovine serum and 1% penicillin/streptomycin in 5% CO_2_ incubator at 37 °C. The next day, the cells were switched to serum-starved medium (DMEM-high-glucose medium supplemented with 1% penicillin/streptomycin). 24 h After serum starvation, cells were treated with T3 (150 nM), T3 (150 nM) + Wortmannin (10 μM) and T3 (150 nM) + Bisphenol A (BPA, 50 μM), respectively. The dosage used in this study was determined with reference to a previous publication [41]. After treatment of 24 h or 48 h, cells were harvested for protein and RNA isolation for further analysis. Differentiated C2C12 cells were maintained in DMEM-high-glucose medium supplemented with 20% fetal bovine serum and 1% penicillin/streptomycin and incubated in 5% CO_2_ incubator at 37 °C. After reaching 90-100% confluence, cells were switched to differentiation medium (DMEM-high-glucose medium supplemented with 2% horse serum and 1% penicillin/streptomycin). Differentiation medium was changed daily and after differentiation for 5 days, cells were switched to serum starvation medium. Differentiation was evaluated using immunofluorescence staining with antibody against myosin heavy chain (#MF20, DSHB, Iowa City, IA, USA) in C2C12. After serum starvation for 24 hours, cells were treated with T3 (150 nM), T3 + Wortmannin (10 μM) and T3 + BPA (50 μM) for 24 h. Protein and RNA were isolated for further analysis after treatment.

### 4.6. Immunofluorescence Staining

Differentiated C2C12 cells were grown on cover slips and fixed with ice cold acetone for 10 min at -20 °C. After fixation, cells were washed in PBS with 5min per time for three times and then blocked with 10% normal goat serum for 2 hours. Primary antibody against myosin heavy chain (#MF20, DSHB, Iowa City, IA, USA) was diluted in 10% normal goat serum (1:200) and cells were incubated with primary antibody for overnight at 4 °C. After incubation, cells were washed three times in PBS for 5 min at a time and incubated with Alexa Fluor 555 goat anti mouse secondary antibody (#A32727, ThermoFisher Scientific, Waltham, MA, USA) for 2 hours at room temperature. Cells were washed three times and counterstained with DAPI. After washing, cover slips were mounted with mounting media. Images were obtained with Keyence fluorescent microscopy (Osaka, Osaka, Japan).

### 4.7. Statistics

GraphPad Prism software was used for statistical analysis, and results are expressed as means ± SEM. Statistical significance between multiple groups was determined by one-way (Dunnett’s test for comparison with control group or Tukey’s test for multiple comparison between groups) or two-way (Tukey’s test for multiple comparison between groups) analysis of variance (ANOVA). Statistical significance was set at *p* < 0.05.

## Data Availability

N/A.

## Supplementary Materials

The following are available online at https://www.mdpi.com/1422-0067/22/6/2928/s1.
