# Supplementary material for: RBM20-Mediated Pre-mRNA Splicing Has Muscle-Specificity and Differential Hormonal Responses between Muscles and in Muscle Cell Cultures
Source: Int J Mol Sci. 2021 Mar 13;22(6):2928. doi: 10.3390/ijms22062928 (PMC7999644; doi:10.3390/ijms22062928)
Supplement: Supplementary file 1 [file ijms-22-02928-s001.pdf]

Supplemental Data

Table S1. RT-PCR primers

**Primers**	**Primer sequence**
Rat titin middle Ig	71 F: TCAACGTGGACTCGACAGACATC<br>84 R: CAGAAACCAGCCCACTGATATTG
Rat titin M-line	362-F: TGTTCAGCTACAGCTTCCTT<br>364-R: AGTCAGATCCAAATTCATTCCC
Rat titin Zr 1-3	7-F: ACGGTGTCCAGGAGCAAGTG<br>10-R: TTGGCTTTGGTTGCGGAGA
Rat titin Zr 3-7	10-F: TGCCAAAGGTAGTGATCTCCG<br>14-R: GTGGTCTGCTGAGCATAGGAT
Rat CamKIIδ	F: AAGGGTGCCATCTTGACAAC<br>R: TCGAAGTCCCCATTGTTGAT
Rat CamKIIg	F: CAACGGTCAACAGTGGCATCC<br>R: GTGTAGGCCTCAAAGTCCCCA
Rat Pdlim3	F: GAACTAAGTGGGTTGTCCGGGTTC<br>R: CGATTGTCGTGCAGCATTCGGTAC
Rat Ldb3	F: TCCAAGCGTCCTATCCCCATC<br>R: TGTATTCTGTCCCGGTCATCTG
Rat GAPDH	F: GGTGGACCTCATGGCCTACA<br>R: CTCTCTTGCTCTCAGTATCCTTGCT
Mouse CamKIIδ	F: CGAGAAATTTTTCAGCAGCC<br>R: GTCTTCATCCTCAATGGTGGTG
Mouse CamKIIg	F: AAGGGTGCCATCCTCACAAC<br>R: GTGTAGGCCTCAAAGTCCCCA
Mouse GAPDH	F: GGTGGACCTCATGGCCTACA<br>R: CTCTCTTGCTCAGTGTCCTTGCT

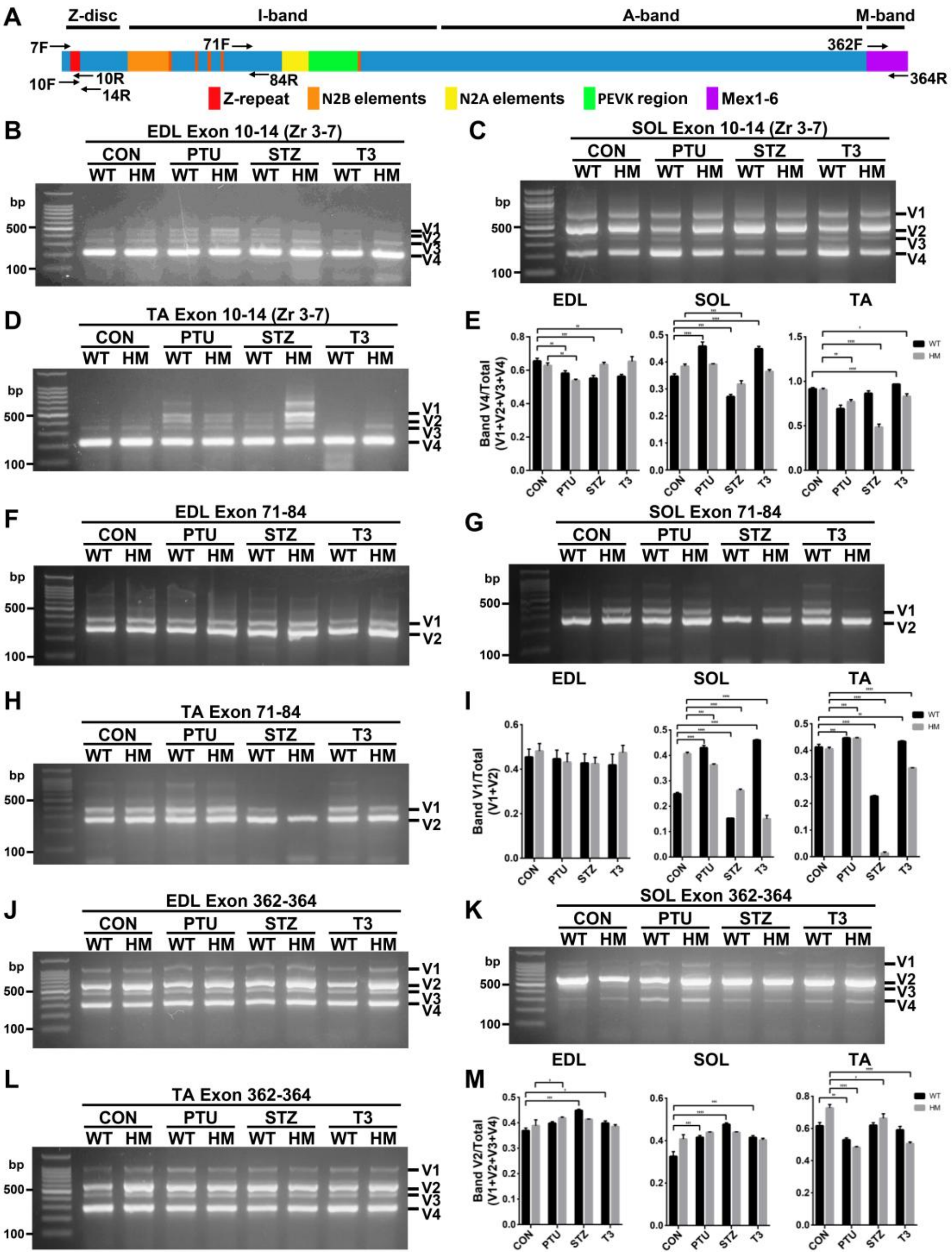

Figure S1. Hormonal effects on alternatively used titin exons in its Z-band, I-band and M-band region of EDL, SOL and TA muscles respectively. (**A**) Schematic structure of titin exons and the primer location in the Z-band, the I-band and the M-Band; (**B–E)** RT-PCR results and quantification of titin alternative exon splicing in the Z-band of TA, SOL and EDL muscles; (**F–I**) RT-PCR results and quantification of titin alternative exon splicing in the I-band of TA, SOL and EDL muscles; (**J–M**) RT-PCR results and quantification of titin alternative exon splicing in the M-band of TA, SOL and EDL muscles; WT, wildtype; HM, RBM20 homozygous knockout; CON, control; PTU, propylthiouracil; STZ, streptozocin; T3, trriodothyronine; TA, tibialis anterior; EDL, extensor digitorum longus; SOL, soleus. Mean ± SEM (n=3), *p<0.05, **p<0.01, ***p<0.001.

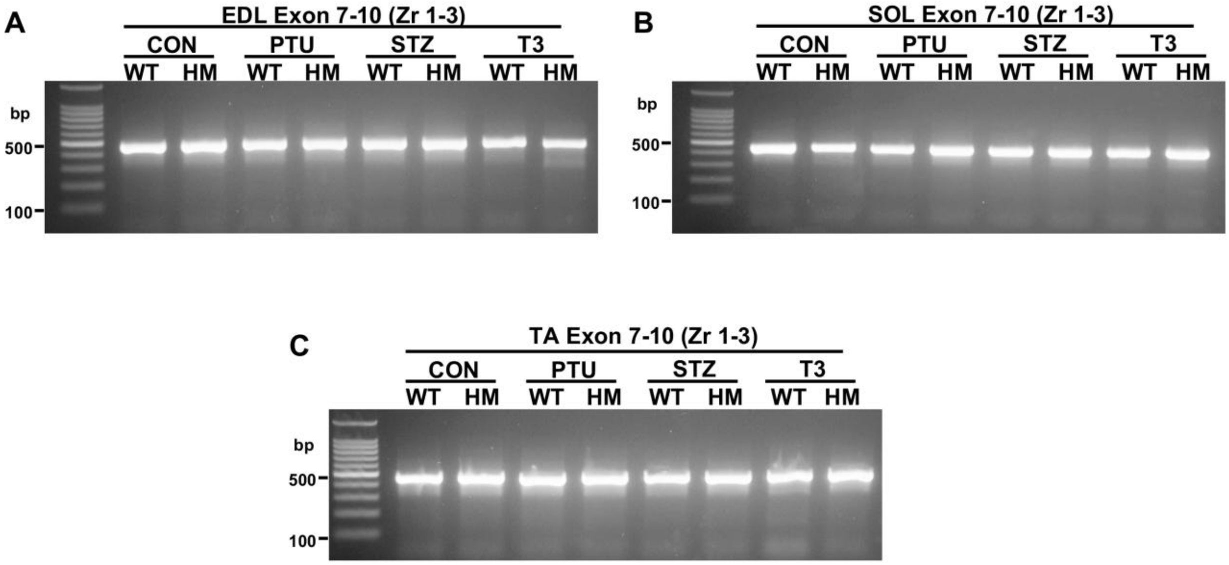

Figure S2. Hormonal effects on titin splicing variants in the Z-band region of TA, EDL and SOL muscles of Wt and Hm rats.**(A–C)** RT-PCR products with primers spanning Zr 1-3 in TA, EDL and SOL muscles of Wt and Hm rats treated with PTU, STZ and T3; WT, wild type; HM, RBM20 homozygous knockout; CON, control; PTU, propylthiouracil; STZ, streptozocin; T3, trriodothyronine; TA, tibialis anterior; EDL, extensor digitorum longus; SOL, soleus; Zr, Z-band repeats.

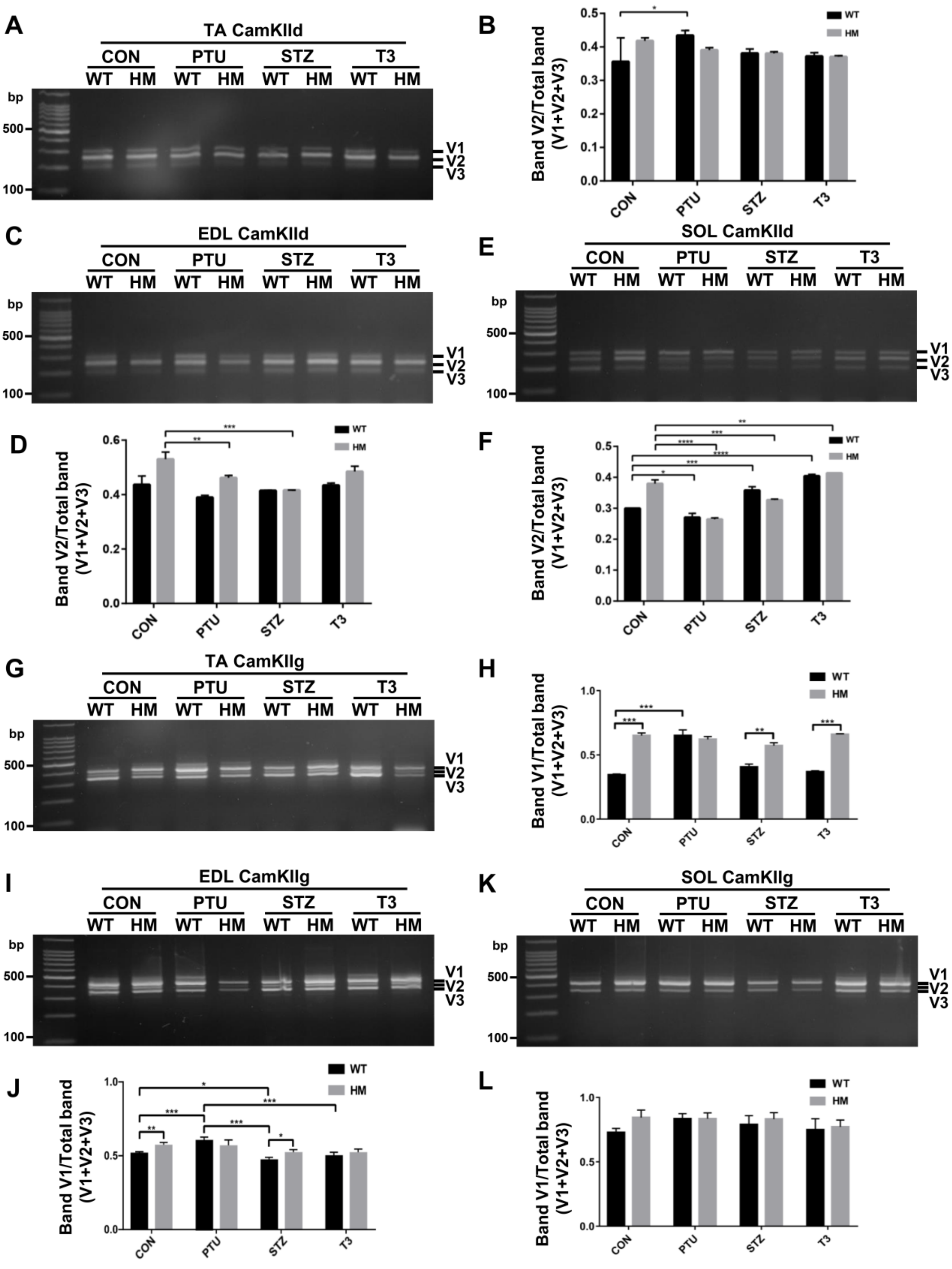

Figure S3. CamKIId and CamKIIg splicing pattern in TA, EDL and SOL muscles of Wt and Hm rats treated with PTU, STZ and T3 respectively. **(A–F)** RT-PCR results and quantification of CamKIId splicing pattern in

TA, EDL and SOL muscles of WT and HM rats treated with PTU, STZ and T3; (**G–L**) RT-PCR results and quantification of CamKIIg splicing pattern in TA, EDL and SOL muscles of WT and HM rats treated with PTU, STZ and T3. WT, wildtype; HM, RBM20 homozygous knockout; CON, control; PTU, propylthiouracil; STZ, streptozocin; T3, trriodothyronine; TA, tibialis anterior; EDL, extensor digitorum longus; SOL, soleus. Mean ± SEM (n=3), *p<0.05, **p<0.01, ***p<0.001.

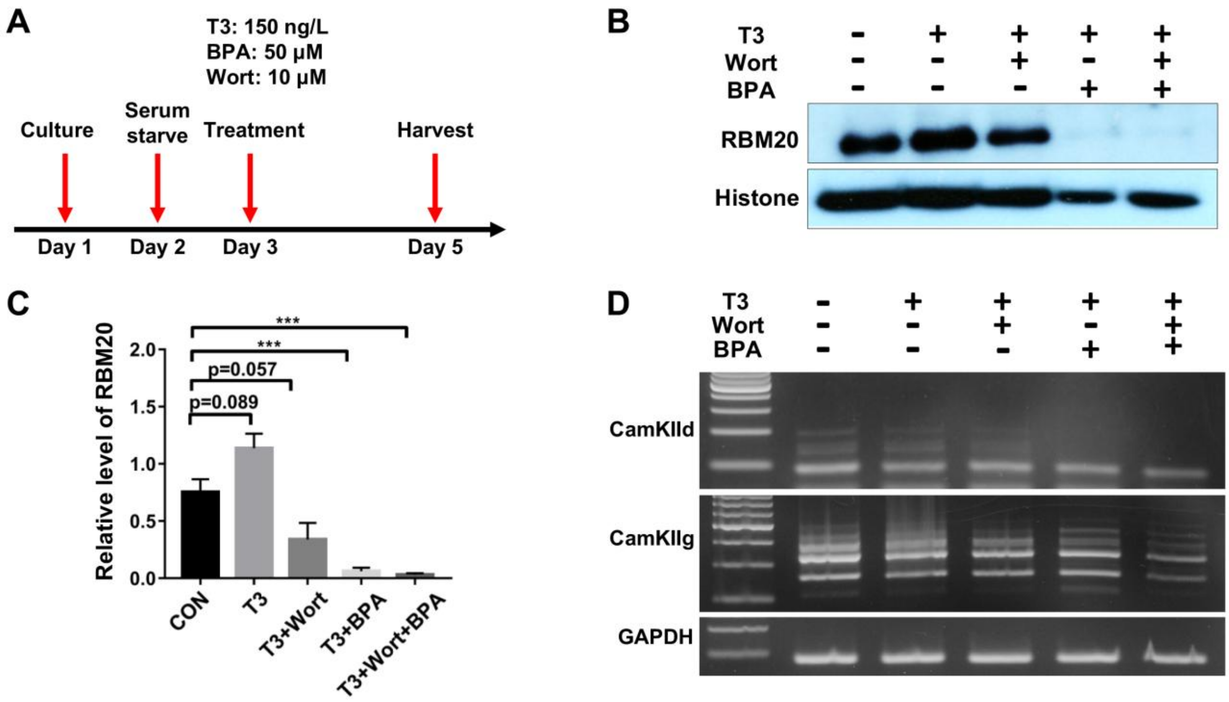

Figure S4. RBM20 expression and pre-mRNA splicing of CamkIId and CamkIIg in undifferentiated C2C12 cells via the genomic and non-genomic pathways. (**A**) Treatment timeline of C2C12 cells with T3, Wort and BPA; (**B**) Western blotting of RBM20 expression in C2C12 cells treated with T3, Wort and BPA respectively; (**C**) Quantification of RBM20 expression level. (**D**) RT-PCR detection of pre-mRNA splicing of CamKIId and CamKIIg genes in C2C12 cells treated with T3, Wort and BPA respectively. CON, control; T3, trriodothyronine; Wort, Wortmannin; BPA, Bisphenol A; Histone, protein loading control; GAPDH, housekeeping gene. Mean±SEM (n=5), ***p<0.001.
